# The gamification of medical education: a broader perspective

**DOI:** 10.3402/meo.v20.30566

**Published:** 2015-12-17

We take great interest in the letter published by Ahmed et al. (1) who discussed the impressive success of gamification, the use of game design elements in non-game context (2), advocating its use in medical education. Despite the hype surrounding gamification (3), this letter provides a broader perspective on the limitations of gamification highlighting common pitfalls to avoid when attempting its implementation.

First, it is important to recognize what gamification can and cannot achieve. Gamification is a process of enhancing services with motivational affordances borrowed from games in order to invoke behavioral outcomes (3). It is therefore imperative to gamify the correct process. Many instructors have made the mistake of trying to gamify an outcome rather than a behavior. For example, you cannot gamify good grades but you can gamify the learning process to motivate students to achieve good grades (4). Even when one has successfully implemented gamification in education, it must be stressed that at its maximum capacity, it can only ever increase motivation and engagement. Sometimes, this may not be enough to achieve academic success; a student may be highly motivated yet still struggle with performance in examinations (4).

Second, when using motivational affordances from games, such as points, rewards, leaderboards, and achievements, implementers need to ensure these motivational affordances resonate with the intrinsic values of the user, otherwise the user may find the gamification meaningless (5). Gamification should be seen as a catalyst to help students carry out specific behaviors they intrinsically find rewarding (6).

Third, implementers must reflect on the outcome of their gamification. Often in efforts to emulate the motivational abilities of games, gamification reduces the complexity of a well-designed game into its simplest components, merely points, leaderboards, and achievements. This reductionist approach does not just result in an experience that fails to engage but can even damage existing interest or engagement (5). Gamification is not a replacement for a well-designed and thoughtful experience. In the context of education, for example, it should not be used to replace face-to-face teaching; indeed it has been shown that retention rates increase when the students are making positive meaningful connections with staff and faculty (7). Furthermore, particularly with the implications of leaderboards, social status may become the motivator, and there is evidence that this can be distasteful for some users (6). This highlights the importance of knowing one's users and the context in which gamification is being used.

Gamification has been successful in many fields, but it has not come without its share of failures (8). Future implementers of gamification in medical education should learn from these failures and avoid the pitfalls discussed. Further research is required into the implementation of gamification in medical education.

*Mohammed Muntasir* School of Medicine Imperial College London London SW7 2AZ, UK Email: mohammed.muntasir11@imperial.ac.uk *Mustafa Franka* School of Medicine Imperial College London London, UK *Bashar Atalla* School of Medicine Imperial College London London, UK *Sarim Siddiqui* School of Medicine Imperial College London London, UK *Umair Mughal* School of Medicine Imperial College London London, UK *Ibtesham T. Hossain* School of Medicine Imperial College London London, UK 
